# Outcomes of normotensive IgA nephropathy patients with mild proteinuria who have impaired renal function

**DOI:** 10.1080/0886022X.2019.1654512

**Published:** 2019-09-13

**Authors:** Min Tan, Jing Fang, Qianqian Xu, Cong Zhang, Guming Zou, Min Wang, Wenge Li

**Affiliations:** Center of Nephrology, China-Japan Friendship Hospital, Health Ministry of China, Beijing, China

**Keywords:** IgA nephropathy, proteinuria, hypertension, renal failure, pathology

## Abstract

**Purpose:** Typically, IgA nephropathy is a slowly progressive type of glomerulonephritis. High-grade proteinuria and hypertension are predictors of reduced kidney function. However, we found some normotensive patients with mild proteinuria could exhibit impaired renal function at the time of IgAN diagnosis. We therefore conduct a study to highlight the occurrence of these cases and to define their clinical characteristics and outcomes.

**Methods:** The clinical, laboratory, pathological manifestations and outcomes of these IgAN patients were collected and were compared with normotensive IgA nephropathy patients with mild proteinuria and normal renal function. Patients were analyzed according to different pathological characteristics. Survival curves were constructed according to the Kaplan–Meier method.

**Results:** Of all normotensive IgA nephropathy patients with mild proteinuria, 108 (10.1%) patients had impaired renal function. Ischemic sclerosis (79 patients) and fibrous crescent (25 patients) were the main pathological characteristics. Macroscopic hematuria (1.3%), prodromal infection (13.9%) and high serum IgA (11.4%) were significantly lower prevalences, but only proteinuria (26.6%) was more common in ischemic sclerosis group patients. Only hematuria were not found in ischemic sclerosis group and crescent group patients. The median follow-up were about 5 years. Patients in crescent group had a poor outcome compared with patients in ischemic sclerosis group.

**Conclusions:** Some normotensive IgA nephropathy patients with mild proteinuria had impaired renal function at diagnosis. Ischemic sclerosis and fibrous crescent were the main pathological features in these patients. Patients in the crescent group had a worse outcome than patients in the ischemic sclerosis group.

## Introduction

Immunoglobulin A nephropathy (IgAN), or Berger’s disease, is the most common type of primary glomerulonephritis worldwide [1]. Although this was initially regarded as a benign condition, more recent studies with long-term follow-up have revealed that the development of progressive renal failure is frequent [2–5]. Typically, IgAN is a slowly progressive type of glomerulonephritis, and most patients exhibit normal kidney function at the time of diagnosis. Patients with IgAN have a variable clinical course, such that 6–43% progress to end-stage renal disease over 10 years [6–8]. Previous studies have identified clinical features, such as high-grade proteinuria and hypertension, as predictors of reduced kidney function [3,7–10]. However, these predictors are not consistent. We have noticed that some normotensive patients with mild proteinuria could exhibit impaired renal function at the time of IgAN diagnosis. The clinicopathological features and outcomes of these patients have not been investigated in detail. Therefore, we conducted a retrospective analysis of IgAN patients with the above characteristics.

## Methods

### Study population

**Figure 1.**
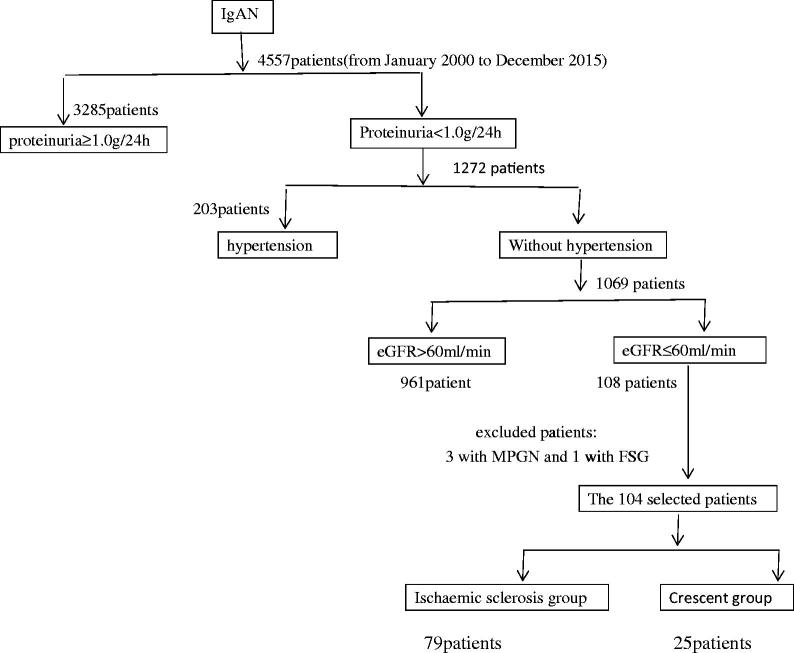
The flow chart of the 104 patients selected out of 1069 original cohort.

In total, 1069 normotensive patients with mild proteinuria were diagnosed with IgAN between January 2000 and December 2015 at the China-Japan Friendship Hospital in Beijing, China. The study sample comprised 108 adult patients from among the 1069 patients (10.1%). Inclusion criteria (Figure 1) were proteinuria <1.0 g/24 h (the mean of three 24-h proteinuria measurements before kidney biopsy), absence of hypertension (systolic blood pressure <140 mmHg and diastolic blood pressure <90 mmHg, without antihypertensive drugs), and impairment of renal function (estimated glomerular filtration rate [eGFR] ≤ 60 mL/min/1.73 m^2^). Idiopathic IgAN was diagnosed based on the presence of predominant mesangial IgA deposits by immunofluorescence and electron microscopy. Patients with Henoch-Schönlein purpura, liver disease, diabetes, systemic disease, or any type of secondary IgAN were excluded. Patients diagnosed with IgAN combined with tubulointerstitial nephritis were excluded. The control group comprised 100 IgAN patients who were diagnosed during the same period with proteinuria <1.0 g/24 h, normal renal function (eGFR > 60 mL/min/1.73 m^2^), and absence of hypertension.

### Data collection

Clinical and laboratory data recorded at biopsy included gender, age, medical history, presenting symptoms, medications, blood pressure, 24-h urinary protein excretion, serum creatinine (Scr), estimated GFR (eGFR, calculated using MDRD equation) and Serum IgA.

### Renal biopsy

All renal biopsy specimens were reviewed by a single pathologist who was blinded to the patients’ clinical conditions. Renal biopsies of ≥10 glomeruli were collected and processed for light and immunofluorescence microscopy.

In light microscopy, renal lesions were analyzed according to pathological schema described previously [8,11,12]. (1) The percentages of glomeruli with cellular crescents, fibrocellular crescents, fibrous crescents, ischemic sclerosis, nonischemic global sclerosis and segmental sclerosis to total glomeruli number. (2) Mesangial proliferation index (MsI): no or focal mild proliferation, 1+; diffuse mild or focal prominent proliferation, 2+ and diffuse prominent proliferation, 3+. (3) Endothelial proliferation index (EPI): *<*50% of glomeruli proliferated, 1+; *>*50% of glomeruli proliferated but most were segmental, 2+ and *>*50% of glomeruli globally proliferated, 3+. (4) Tubulointerstitial index (TI): according to the ratio of involved interstitial fibrosis area to total tubulointerstitial area in the slide (0%, 0; *<*10%, 1+; 10–25%, 2+; 25–50%, 3+; 50–75%, 4+; ≥75%, 5+). (5) Renal arterial or arteriolar wall thickening was graded according to the cross-sectional ratio of luminal diameter to outer diameter: grade 1, more than half; grade 2, one-half to one-third; grade 3, one-third to one-quarter; grade 4, one-quarter or less. A mild arterial lesion was defined as grade 1–2, and a severe arterial lesion was defined as grade3-4, or with hyaline change in any grade. We also assessed the histologic findings according to Oxford classification criteria and categorized them with independent predictors which were (1) mesangial hypercellularity (M), (2) segmental glomerulosclerosis (S), (3) endocapillary hypercellularity (E), and (4) tubularatrophy/interstitial fibrosis (T) [13].

### Follow-up and study endpoint

All patients were scheduled for 3-month follow-up visits in the clinic. At each visit, medication use and laboratory data were recorded. Two patients in the impaired renal function group were lost to follow-up within 12 months after renal biopsy and were therefore excluded from the study. All 100 patients with normal renal function had a complete follow-up record. The study endpoint was a doubling of the baseline serum creatinine concentration, defined as a sustained >2-fold increase in serum creatinine levels for at least three consecutive measurements.

### Statistical analysis

Quantitative data were expressed as means ± standard deviations or means (minimums—maximums). Quantitative parameters were compared between groups by using t-tests. Qualitative variables were described by frequency distribution and were compared between groups by the chi-squared test. The cumulative probability of developing a defined clinic event was estimated using the Kaplan–Meier method, and survival curves were compared using log-rank tests. Multivariate logistic regression analysis was used to identify independent factors associated with the development of the endpoint criterion. *p* < .05 was considered statistically significant. All statistical analyses were performed using SPSS statistical software (version 12.0; SPSS, Inc., Chicago, IL, USA).

## Results

Pathological analysis revealed that ischemic sclerosis and crescent formation were the primary pathological characteristics in IgAN patients. Based on their pathological characteristics, we divided patients with impaired renal function into two groups: patients in which >20% of glomeruli exhibited ischemic sclerosis were placed in the ischemic sclerosis group (79 patients), and patients in which >20% of glomeruli exhibited crescent formation were placed in the crescent group (25 patients). Three patients who had mesangial proliferative glomerulonephritis combined with red blood cell casts were excluded. One patient who had a pathological background of segmental glomerulosclerosis was excluded. The remaining 104 patients wre the object we studied.

### Baseline clinical and laboratory features

There were more men in the ischemic sclerosis group than in the control group of IgAN patients with normal renal function (75.9% vs. 56.0%, *p* < .05). No patients in the ischemic sclerosis or crescent groups had only hematuria (isolated microscopic hematuria). Patients in the ischemic sclerosis group had significantly lower prevalences of macroscopic hematuria (1.3% vs. 32.0% and 39.0%, *p* < .01) and prodromal infection (13.9% vs. 40.0% and 37.0%, *p* < .01). The ischemic sclerosis group included fewer patients with elevated serum IgA than the crescent group (11.4% vs. 36.0%, *p* < .05). Only proteinuria (26.6% vs. 0% [crescent group] and 9.0% [IgAN with normal renal function group], *p* < .01) and severe arterial lesion (74.7% vs. 40.0% [crescent group] and 32.0% [IgAN with normal renal function group], *p* < .01) were more common among patients in the ischemic sclerosis group. Patients in the ischemic sclerosis group had higher levels of serum uric acid (vs. crescent group, *p* < .05; vs. IgAN with normal renal function group, *p* < .01). The three populations of IgAN patients did not differ in terms of mean age, pre-biopsy history, proteinuria, or blood pressure (Table 1).

**Table 1.** Comparison of clinical features between IgAN patients in ischemic sclerosis group, crescent group and normal renal function group.

	Ischemic sclerosis group (*n* = 79)	Crescent group (*n* = 25)	IgAN with normal renal function (*n* = 100)
Male gender (N[%])	60 (75.9%)^a^*	16 (64.0%)	56 (56.0%)
Age, years (mean[SD])	31.6 ± 15.9	32.1 ± 9.7	31.8 ± 5.4
Pre-biopsy history (mean[SD])	13.1 ± 8.2	10.6 ± 6.7	11.5 ± 11.3
Macroscopic hematuria (N[%])	1 (1.3%)^a,b^**	8 (32.0%)	39 (39.0%)
Only Hematuria (N[%])	0	0	24 (24.0%)^b,c^**
Only Proteinuria (N[%])	21 (26.6%)^a,b^**	0	9 (9.0%)
Prodromal infection (N[%])	11 (13.9%)^a,b^**	10 (40.0%)	37 (37.0%)
Systolic BP (mmHg)	117 ± 41.0	123 ± 38.6	117 ± 45.0
Diastolic BP (mmHg) 69 ± 31	74 ± 36	70 ± 31	
SCr (μmol/L, mean[SD])	140 ± 57	142 ± 88	73 ± 20^b,c^**
eGFR (ml/min)	55.3 ± 5.3	53.5 ± 9.8	90.8 ± 20.1^b,c^**
Proteinuria (g/24 h)	0.53 ± 0.35	0.60 ± 0.32	0.54 ± 0.28
Serum Uric Acid (mmol/L)	455 ± 116^b^*	430 ± 80	365 ± 98^b,c^**
Serum IgA higher than normal (N,%)	9 (11.4%)^b^*	9 (36.0%)	23 (23.0%)

BP: blood pressure; Scr: serum creatinine; eGFR: estimated GFR.

*P < 0.05, **P < 0.01. ^a^versus IgAN with normal renal function group. ^b^versus crescent group; ^c^versus ischemic sclerosis group.

### Histologic findings

Pathological analysis (Table 2) revealed that patients in the ischemic sclerosis group had the highest prevalences of ischemic sclerosis (30.7% vs. 6.37% [crescent group] and 2.83% [IgAN with normal renal function group], *p* < .01) and severe arterial lesion (74.7% vs. 40.0% [crescent group] and 32.0% [IgAN with normal renal function group], *p* < .01). The prevalence of crescent formation (31.7% vs. 0.31% [ischemic sclerosis group] and 1.15% [IgAN with normal renal function group], *p* < .01) was significantly higher in patients in the crescent group. Notably, most of the crescents were fibrous crescents. According to the Oxford classification, T0 was the main tubulointerstitial manifestation, while T1 was more common in the ischemic sclerosis (17.7%) and crescent groups (20.0%) than in IgAN patients with normal renal function (2.0%). S1 was observed in 40% of patients in the crescent group; this prevalence was significantly higher than that in the ischemic sclerosis (26.6%) and normal renal function groups (13.0%). No significant differences were observed in the severity of non-ischemic global sclerosis, mesangial proliferation, or endothelial proliferation.

**Table 2.** Comparison of pathological features between IgAN patients in ischemic sclerosis group, crescent group and normal renal function group.

	Ischemic sclerosis group (*n* = 79)	Crescent group (*n* = 25)	IgAN with normal renal function (*n* = 100)
Pathological features			
Non-ischemic global sclerosis (%)	3.0 (0–25.0)	3.77 (0–16.7)	2.02 (0–15.4)
Ischemic sclerosis (%)	30.7 (20.0–75.0)^a,b^**	6.37 (0–18.2)	2.83 (0–18.2)
Segmental sclerosis (%)	1.54 (0–36..4)	3.15 (0–9.7 )	1.35 (0–10.0)
Crescents (%)	0.31 (0–6.67)	31.7 (20–41.9)^a,c^**	1.15 (0–15.8)
Cellular crescents (%)	0	0	0
Fibrocellular crescents (%)	0	2.64 (0–22.2)	0.73 (0–12.5)
Fibrous crescents (%)	0.31 (0–6.67)	29.1 (20–41.9)^a,c^**	1.43 (0–15.8)
MSI (mean[SD])	1.11 (1.0–2.0)	1.36 (1.0–2.0)	1.24 (1.0–2.0)
EPI (mean[SD])	1.01 (0–2.0)	1.09 (0–2.0)	1.01 (1.0–2.0)
TI (mean[SD]	1.39 (0–3.0)	1.60 (0–3.0)	1.04 (0–3.0)^b,c^*
Renal arteriole lesions			
Mild arterial lesion (N,%)	20 (25.3%)^a,b,^**	15 (60.0%)	68 (68.0%)
Severe arterial lesion (N,%)	59 (74.7%)^a,b,^**	10 (40.0%)	32 (32.0%)
Oxford classification			
M1	37 (46.8%)	12 (48.0%)	45 (45.0%)
E1	8 (10.1%)	6 (24.0%)	10 (10.0%)
S1	21 (26.6%)	10 (40.0%)^a^*	13 (13.0%)
T			
T0	65 (82.3%)	20 (80.0%)	98 (98.0%)^b,c^*
T1	14 (17.7%)	5 (20.0%)	2 (2.0%)^b,c^*
T2	0 (0%)	0 (0%)	0 (0%)

MSI: Mesangial proliferation index; EPI: Endothelial proliferation index; TI: Tubulointerstitial index; SD: standard deviation.

*P < 0.05, **P < 0.01. ^a^versus IgAN with normal renal function group. ^b^versus crescent group; ^c^versus ischemic sclerosis group.

### Follow-up and treatment

The median follow-up periods were 61 months (range, 14–109 months) in the ischemic sclerosis group, 58 months (range, 15–110 months) in the crescent group, and 51 months (range, 13–100 months) in IgAN patients with normal renal function. Patients with proteinuria levels >0.3 g/24 h received treatment with either angiotensin-converting enzyme inhibitors or angiotensin receptor blockers. At the final follow-up, a doubling of serum creatinine was observed in 4 patients (5.2%) in the ischemic sclerosis group, 6 patients (24.0%) in the crescent group, and 2 patients (2.0%) in the group with normal renal function. Corticosteroid and/or cyclophosphamide treatment was administered in 1 patient (1.3%) in the ischemic sclerosis group, 4 patients (16.0%) in the crescent group, and 3 patients (3.0%) in the group with normal renal function. The number of patients who received corticosteroid treatment and the incidence of patients who reached the endpoint were highest in the crescent group. At the time of biopsy, blood pressure and proteinuria did not differ among the three groups. At the final follow-up, there were more patients with new-onset hypertension in the ischemic sclerosis (25, 32.5%) and crescent groups (6, 24.0%). The crescent group had higher proteinuria and lower eGFR than the other two groups (Table 3).

**Table 3.** The data of patients at last follow-up in different groups.

	IgAN with impaired renal function		IgAN with normal
	Ischemic sclerosis group (*n* = 77)	Crescent group (*n* = 25)	renal function (*n* = 100)
Follow-up time (months)	61	58	51
At time of biopsy			
Hypertension (*N*, %)	0	0	0
Proteinuria (g/24h)	0.67 ± 0.31	0.68 ± 0.32	0.47 ± 0.28
Scr (μmol/L)	140 ± 52	147 ± 88	80 ± 20
eGFR (ml/min)	52.5 ± 7.3	51.7 ± 5.9	92.8 ± 27.1
Last follow up			
Hypertension (*N*, %)	25 (32.5%)^a^*	6 (24.0%)^a^*	8 (8.0%)
Proteinuria (g/24h)	0.78 ± 0.21	1.30 ± 0.32^a,b^*	0.61 ± 0.30
Scr (μmol/L)	160 ± 48	196 ± 98^a^**^,b^*	83 ± 20
eGFR (ml/min)	47.5 ± 8.3	37.7 ± 10.0^a^**^,b^*	91.5 ± 31.2
Doubling of Scr (*N*, %)	4 (5.2%)	6 (24.0%)^a^**^,b^*	2 (2.0%)
Corticosteroid treatment (*N*,%)	1 (1.3%)	4 (16.0%)^a,b^*	3 (3.0%)

Scr: serum creatinine; eGFR: estimated GFR.

*p < 0.05, **p < 0.01. ^a^versus IgAN with normal renal function group. ^b^versus ischemic sclerosis group.

**Figure 2.**
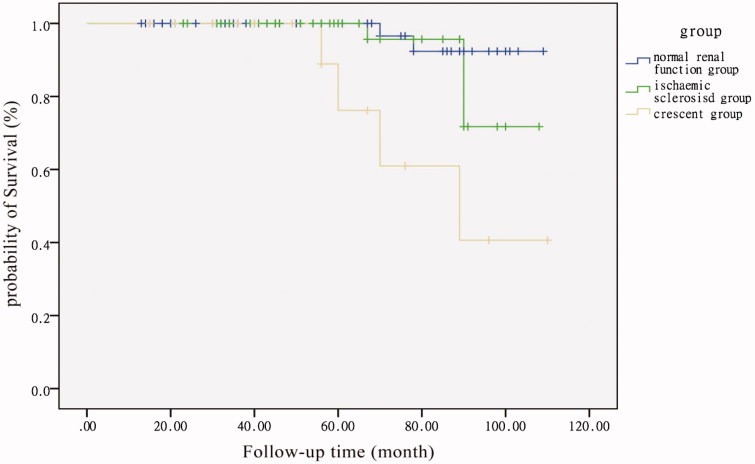
Kidney survival curve in IgAN patients with normal renal function and the two subgroups of IgAN patients with impaired renal function. The primary endpoint was a doubling of the primary baseline serum creatinine. Patients in crescent group had a poor outcome compared with patients in normal renal function (*P* < 0.001, log-rank test) and patients in ischemic sclerosis group (*P* = 0.015, log-rank test).

In Kaplan–Meier analysis (Figure 2), censored for the primary endpoint of the doubling of baseline serum creatinine, patients in the crescent group had a worse outcome than patients in the normal renal function (*p* < .001, log-rank test) and ischemic sclerosis groups (*p* = .015, log-rank test). Multivariate logistic regression analysis was used to identify independent factors for the development of endpoint. The following variables were considered: gender, age, proteinuria, macroscopic hematuria, hypertension, Scr, segmental glomerulosclerosis, ischemic sclerosis, fibrous crescents, ACEI or ARB treatment and corticosteroid treatment. The following parameters are independent risk factors for the doubling of serum creatinine level: segmental glomerulosclerosis (RR 2.56, 95% CI 1.35–9.88, *p* < .01), ischemic sclerosis (RR 1.9, 95% CI 1.52–4.78, *p* = .02) and fibrous crescents (RR 0.51, 95% CI 0.21–0.88, *p* = .005).

## Discussion

IgAN is a highly heterogeneous disease with variable clinical patterns, morphologic variables, long-term renal progression, and geographic prevalence [1,14–18]. Typically, IgAN is a slowly progressive type of glomerulonephritis, and most patients exhibit normal kidney function at the time of diagnosis [10]. High-grade proteinuria (> 1.0 g/24 h) and arterial hypertension have been identified as independent risk factors for progression toward renal failure [3,6,9,19]. However, these risk factors are inconsistent. Our study population included 108 normotensive patients with mild proteinuria who had impaired renal function at the time of IgAN diagnosis. These 108 patients comprised 10.1% of all normotensive patients with mild proteinuria who were diagnosed with IgAN during the study period. This finding suggests that normotensive patients commonly exhibit mild proteinuria and impaired renal function at the time of IgAN diagnosis. Based on their different pathological characteristics, we divided patients with impaired renal function into two groups: ischemic sclerosis and crescent groups. Compared with patients in the crescent and normal renal function groups, patients in the ischemic sclerosis group had unique clinical features: no patients in the ischemic sclerosis group had hematuria alone. Patients in the ischemic sclerosis group also had significantly lower prevalences of macroscopic hematuria and prodromal infection, and were more likely to have proteinuria alone and also to have higher levels of serum uric acid. Elevated serum IgA is a characteristic of IgAN patients. The percentage of IgAN patients with an elevated serum IgA level differs among countries, with a range of 30–70%. We found that fewer patients had elevated serum IgA in the ischemic sclerosis group than in the crescent group (11.4% vs. 36.0%, *p* = .016).

Pathology analysis revealed that ischemic sclerosis and crescent formation were the main pathological characteristics of the IgAN patients in this study. While most crescents were fibrous crescents, more patients (79, 76.0%) had pathological manifestations of ischemic sclerosis. Consistent with the findings of some previous investigations [12,20,21], intrarenal arterial lesions were commonly found in renal biopsies of IgAN patients in our study. Severe arterial lesions were most commonly found in patients in the ischemic sclerosis group (74.7% vs. 40.0% [crescent group] and 32.0% [IgAN with normal renal function group], *p* < .01); this finding suggests that some renal arterial lesions in IgAN patients were not caused by hypertension. Hernández et al. reported finding increased levels of plasma von Willebrand factor in some IgAN patients, even those with normal blood pressure, normal renal function, and absence of proteinuria [22]. Zhang found that intrarenal arterial lesions in patients with IgAN were associated with endothelial cell damage, and that von Willebrand factor levels could serve as an important serological biomarker of severe intrarenal arterial damage. Anti-endothelial cell antibodies, particularly IgG isotypes of these antibodies, may play an important role in the pathogenesis of intrarenal arterial damage in IgAN [12]. The mechanism underlying the formation of intrarenal arterial lesions of IgAN requires further investigation. According to the Oxford classification, T1 was more common in the ischemic sclerosis (17.7%) and crescent groups (20.0%) than in IgAN patients with normal renal function (2.0%). Notably, S1 was observed in 40% of patients in the crescent group. No significant differences were found in the severities of non-ischemic global sclerosis, mesangial proliferation, or endothelial proliferation.

The median follow-up period was approximately 5 years for all three groups. At the final follow-up, the crescent group included more patients who received corticosteroid treatment (4, 16.0%) and a higher incidence of patients who reached the endpoint (6, 24.0%). At the final follow-up, the ischemic sclerosis and crescent groups included more patients with new-onset hypertension (25, 32.5% and 6, 24.0%, respectively) than the group with normal renal function. The crescent group had higher proteinuria and lower eGFR levels than those in the other two groups. Pathological features have been reported as risk factors for the progression of IgAN. Interstitial fibrosis, glomerular sclerosis, and crescent formation have been associated with poor prognosis [4,23–26]. Our study revealed that patients in the crescent group had a worse outcome than patients in the ischemic sclerosis group, suggesting that different types of glomerulosclerosis may have different prognostic effects on IgAN patients. Multivariate logistic regression analysis revealed that segmental glomerulosclerosis, ischemic sclerosis, and fibrous crescents were independent risk factors for the doubling of serum creatinine level.

In conclusion, some normotensive IgA nephropathy patients with mild proteinuria had impaired renal function at diagnosis. Ischemic sclerosis and fibrous crescent were the main pathological features in these IgAN patients. Patients in the crescent group had a worse outcome than patients in the ischemic sclerosis group.
